# Supplementary material for: Highly efficient CRISPR-Cas9-mediated gene knockout in primary human B cells for functional genetic studies of Epstein-Barr virus infection
Source: PLoS Pathog. 2021 Apr 15;17(4):e1009117. doi: 10.1371/journal.ppat.1009117 (PMC8078793; doi:10.1371/journal.ppat.1009117)
Supplement: S1 Fig — (A) Cellular DNAs were PCR amplified, sequenced and analyzed with the Outknocker webtool. The insertions and deletions in exon 2 of the CD46 gene in WT EBV infected primary human B cells were analyzed 8 days post nucleofection. Reads from B cells nucleofected with the CD46-Cas9 RNP complexes (CD46-Cas9) were aligned to the hg19 reference human genome. The reads are summarized in pie charts and unique sequences are displayed below as examples. The target site of gRNA1, the upstream CD46 specific crRNA is highlighted in yellow within the reference sequence of the CD46 locus; the PAM sequence is highlighted in orange. (B) Representative agarose gels of PCR products encompassing exon 2 of the CD46 locus in non-infected and EBV-infected B cells after nucleofection with two CD46-Cas9 RNP complexes at indicated time point post nucleofection. The expected PCR product of the intact CD46 locus is 276 bp in length. PCR products obtained from the CD46 locus with the partially deleted exon 2 are about 178 bp in length. Experiments shown in panel B are representative of three independent biological replicates from non-infected and EBV infected cells. (PDF) [file ppat.1009117.s001.pdf]

A

MiSeq sequencing and data analysis of CD46-Cas9 cells and controls

Outknocker web tool

CD46 Cas9 cells:
Knockout efficiency: 84.2 %

WT cells:
Knockout efficiency: 0.1 %

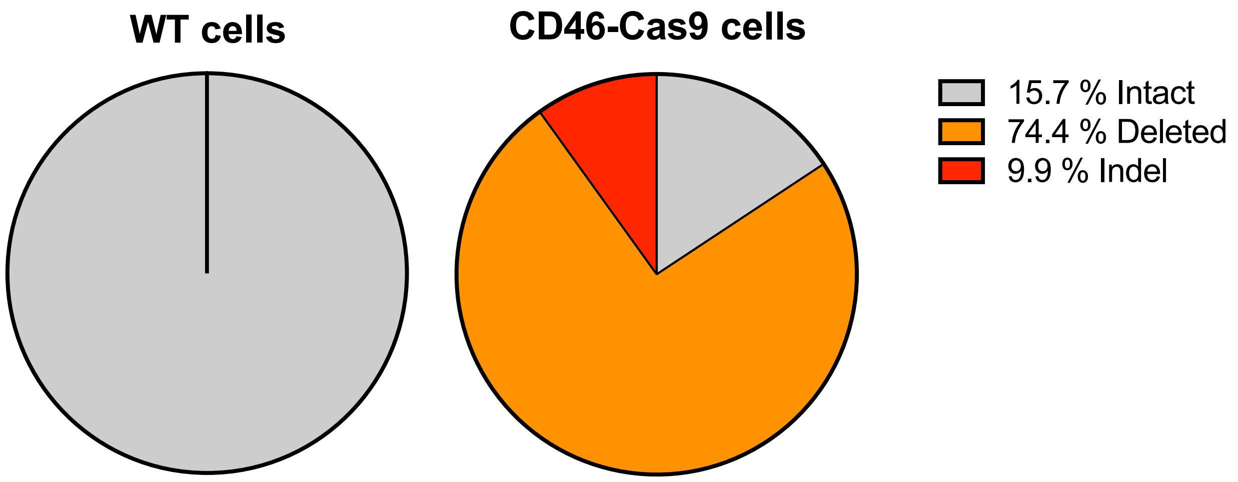

	PAM	
REFERENCE	CTATGGAGCTCATTGGTAAACCAAAACCCTACTATGAGATTGGTGAACGAGTAGATTATAAGTGTAAAAAAGGATACTTC	
Intact		**15.71%**
no indel	CTATGGAGCTCATTGGTAAACCAAAACCCTACTATGAGATTGGTGAACGAGTAGATTATAAGTGTAAAAAAGGATACTTC	919 reads
Deleted		**74.35%**
90nt deletion	CTATGGAGCTCATTGGTAAACCAAAACCCTACTAT	274 reads
91nt deletion	CTATGGAGCTCATTGGTAAACCAAAACCCTACT	2995 reads
92nt deletion	CTATGGAGCTCATTGGTAAACCAAAACCCTACTA	1081 reads
Indel		**9.94%**
1nt deletion	CTATGGAGCTCATTGGTAAACCAAAACCCTACT_TGAGATTGGTGAACGAGTAGATTATAAGTGTAAAAAAGGATACTTC	316 reads
2nt deletion	CTATGGAGCTCATTGGTAAACCAAAACCCTACT__GAGATTGGTGAACGAGTAGATTATAAGTGTAAAAAAGGATACTTC	265 reads

B

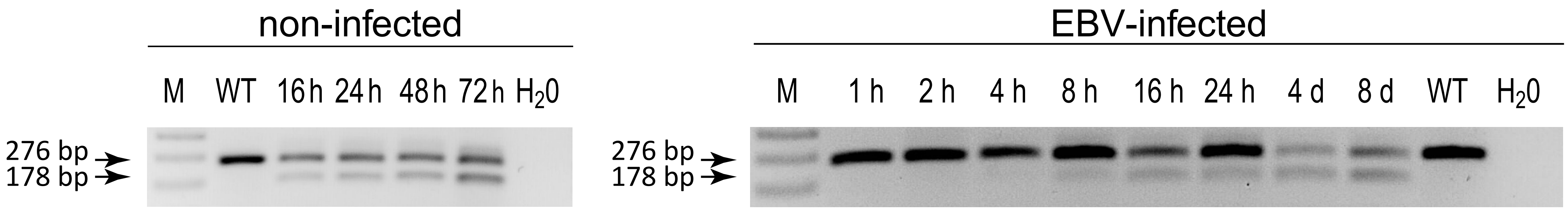

Supporting Figure S1
