# Supplementary figures and images for: Highly efficient CRISPR-Cas9-mediated gene knockout in primary human B cells for functional genetic studies of Epstein-Barr virus infection
Source: PLoS Pathog. 2021 Apr 15;17(4):e1009117. doi: 10.1371/journal.ppat.1009117 (PMC8078793; doi:10.1371/journal.ppat.1009117)

A

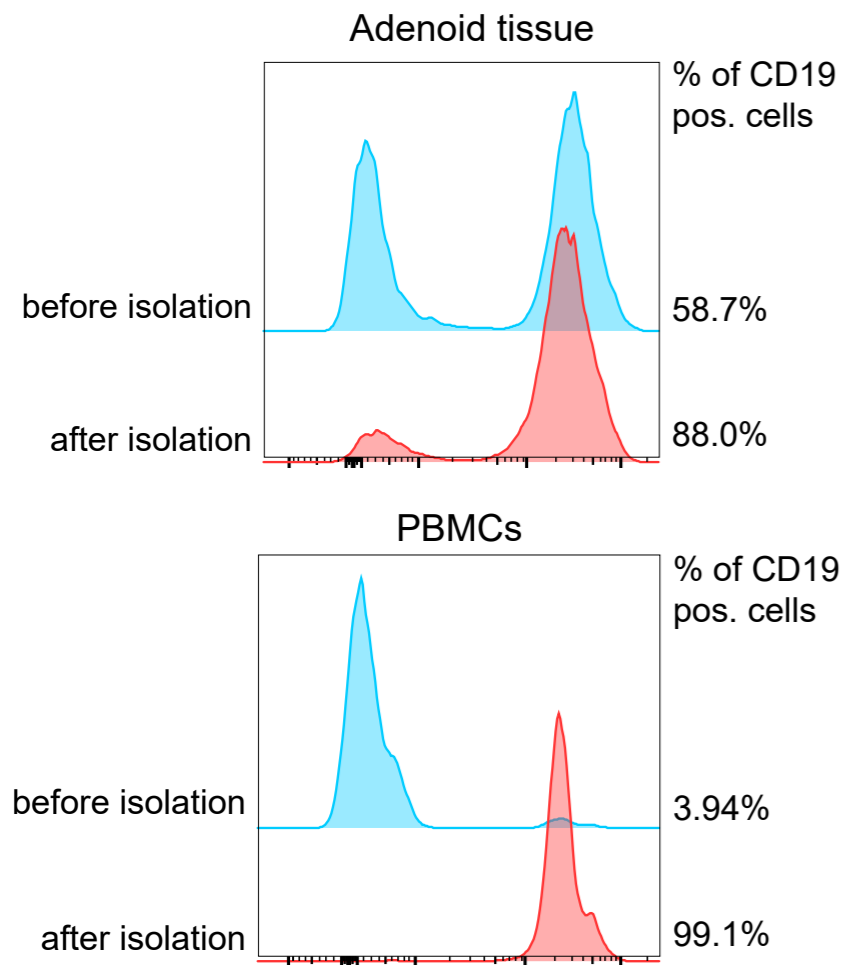

B

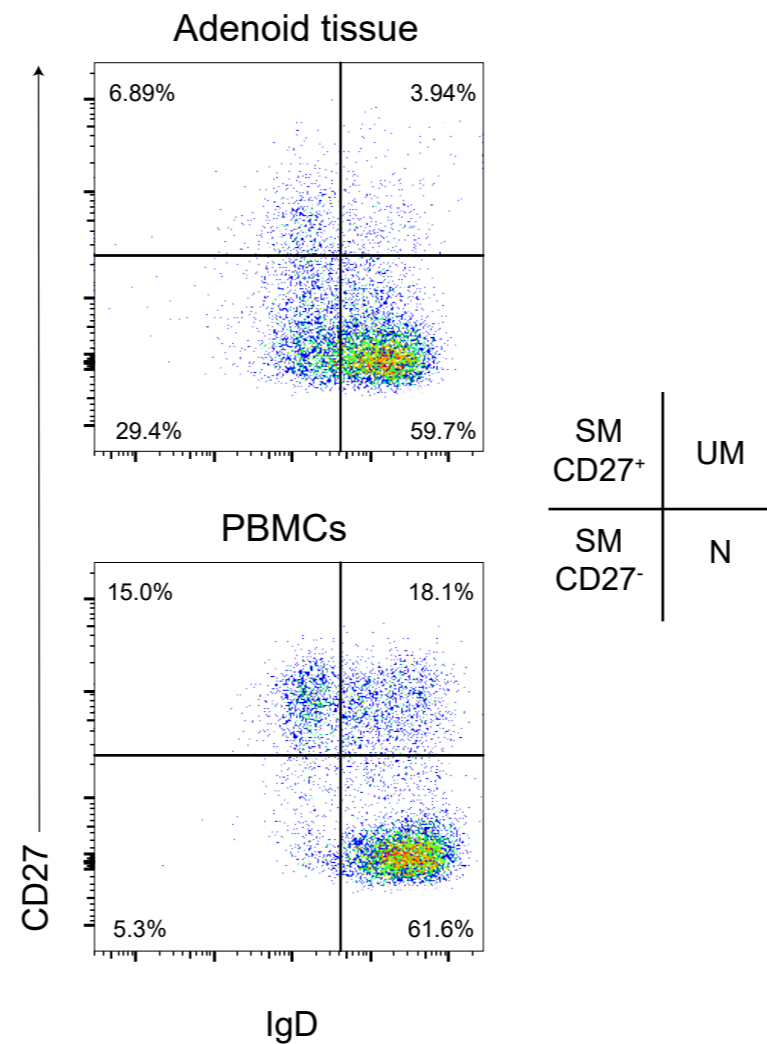

C

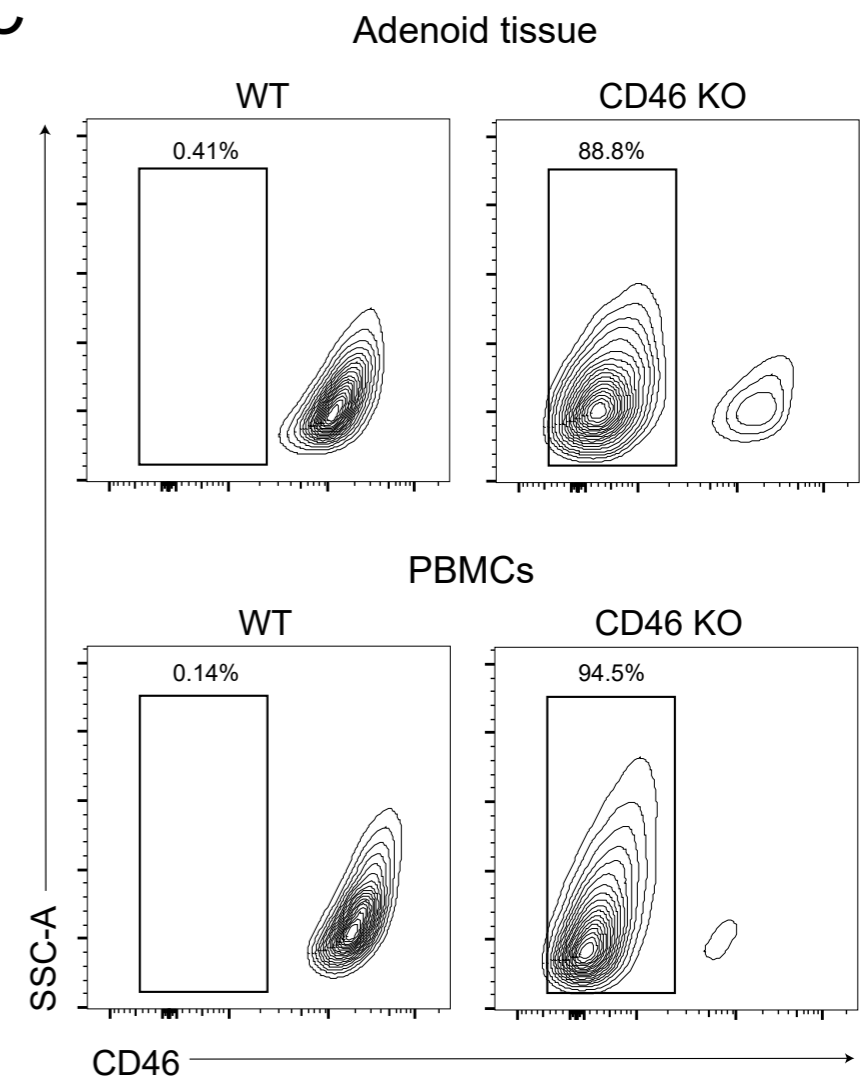

D

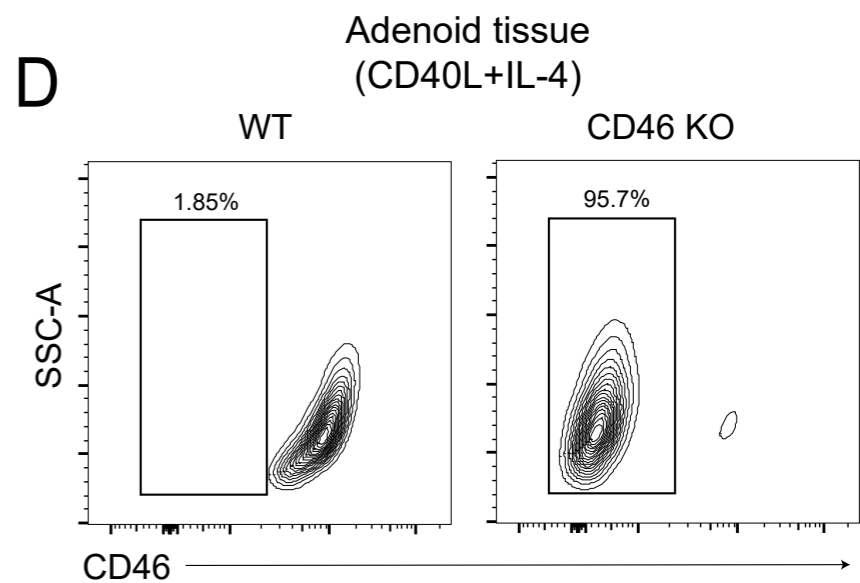

Supplement: S2 Fig — (A) B cell identity after B cells isolation and purification from adenoids or PBMCs (B) Flow cytometric analysis characterizing the different subsets of B cells purified from adenoid tissues or PBMC samples. The nomenclature describing different B cell subsets is depicted. SM, switched memory; N, naïve; UM, unswitched memory. (C) Flow cytometric analysis of CD46 surface levels on B cells obtained from adenoid and PBMC samples one week after nucleofection and infection with EBV. (D) B cells obtained from adenoid tissue were nucleofected with CD46-Cas9 RNP complexes and cultivated on irradiated CD40 ligand (CD40L) feeder cells in the presence of IL-4 for 9 days. As in panel C, the CD46 surface levels are shown. (PDF) [file ppat.1009117.s002.pdf]

A

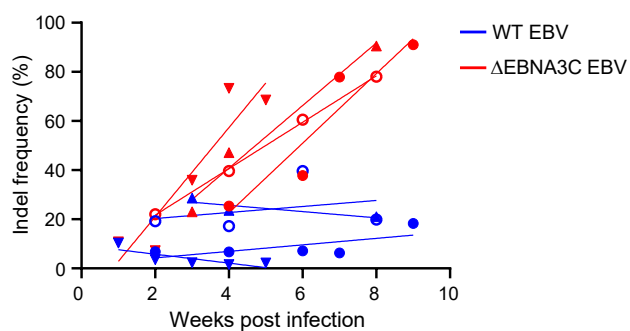

B

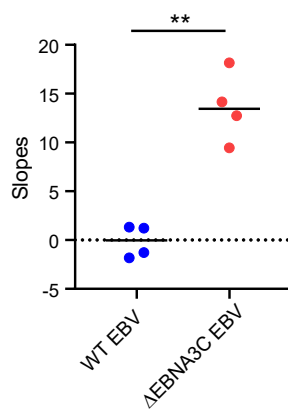

C

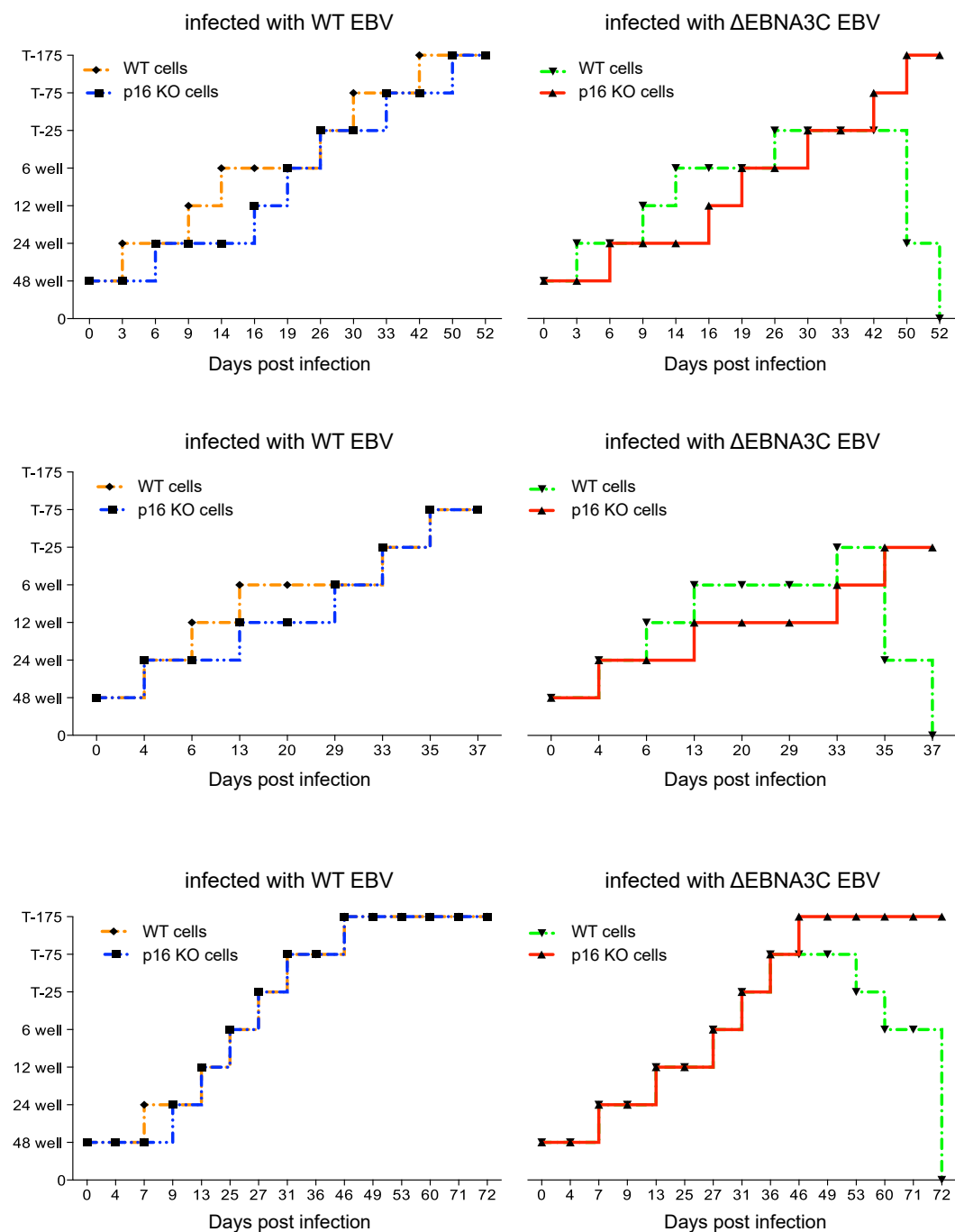

Supplement: S4 Fig — (A) To study the biological effect of the CDKN2A knockout in a time course analysis, p16 WT and p16 KO cells were mixed such that the fraction of the latter was in the order of 10 to 20% prior to infection as described in Fig 4. The graph shows the statistical analysis of the time-related frequencies of CDKN2A negative B cells (according to identified indels) infected with WT or ΔEBNA3C EBV strains from four independent experiments. Exemplary results from two experiments are shown in Fig 4A. Linear regression analysis was performed with data from all four experiments to calculate the slopes of linear regression from each experiment. (B) Slope values were transferred into a column data table. P values were calculated using the unpaired t-test. **, P<0.05. (C) Cell expansion of four different B cell populations were recorded and plotted as a function of days post nucleofection (x-axis) versus the format of the cell culture vessel (y-axis) starting with a single well in a 48-well cluster plate up to T175 cell culture flasks. 2×10^6^ B cells with an intact CDKN2A locus (p16 WT cells) or cells with an edited CDKN2A gene (p16 KO cells) were infected with the WT EBV (left panels) or ΔEBNA3C EBV (right panels). The results were consistent between four additional biological replicates. Three replicates are shown here and one is provided in Fig 4C. (PDF) [file ppat.1009117.s004.pdf]

A

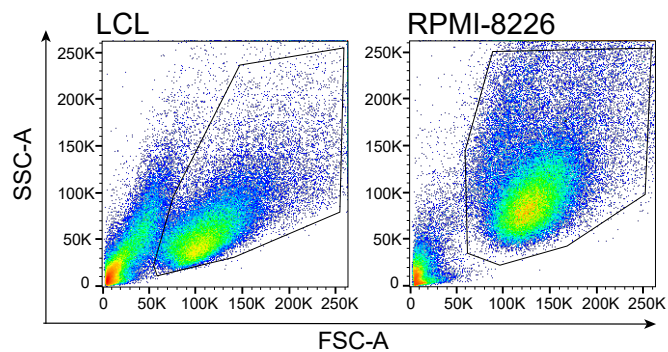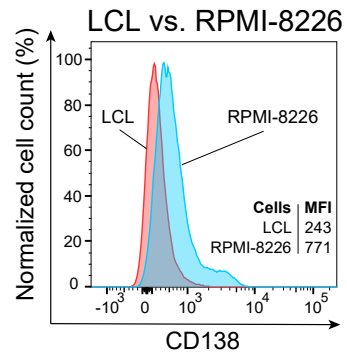

Supplementary Figure S5

Supplement: S5 Fig — The left and middle panels show forward and sideward scatter data of an established EBV-infected lymphoblastoid cell line (LCL) and the RPMI-8226 cell line, respectively. Both cell lines were analyzed with a CD138 specific antibody (Invitrogen, APC-conjugated, #17-1389-42). RPMI-8226 cells (blue) express higher levels of CD138 than the LCL (red) in the graph on the right with normalized counts (y-axis). RPMI-8226 cells also reveal a subpopulation that exhibits a higher level of CD138 surface expression than the majority of the cells. (PDF) [file ppat.1009117.s005.pdf]
