# Supplementary material for: Highly efficient CRISPR-Cas9-mediated gene knockout in primary human B cells for functional genetic studies of Epstein-Barr virus infection
Source: PLoS Pathog. 2021 Apr 15;17(4):e1009117. doi: 10.1371/journal.ppat.1009117 (PMC8078793; doi:10.1371/journal.ppat.1009117)
Supplement: S3 Fig — Cellular DNA of the CDKN2A locus was PCR amplified, sequenced and analyzed with the Outknocker webtool. The insertions and deletions in exon 1α of CDKN2A encoding p16INK4a were analyzed in primary human B cells two weeks post nucleofection. Reads for WT and p16 KO cells were aligned to the hg19 reference human sequence. The results are summarized in pie charts and unique read sequences are displayed below as examples. The target site of the CDKN2A specific crRNA and the PAM sequence are highlighted in yellow and orange, respectively, in the reference sequence. (PDF) [file ppat.1009117.s003.pdf]

MiSeq sequencing and data analysis of p16 KO cells and controls

Outknocker web tool

p16 KO cells:
Knockout efficiency: 74.6 %

WT cells:
Knockout efficiency: 0.1 %

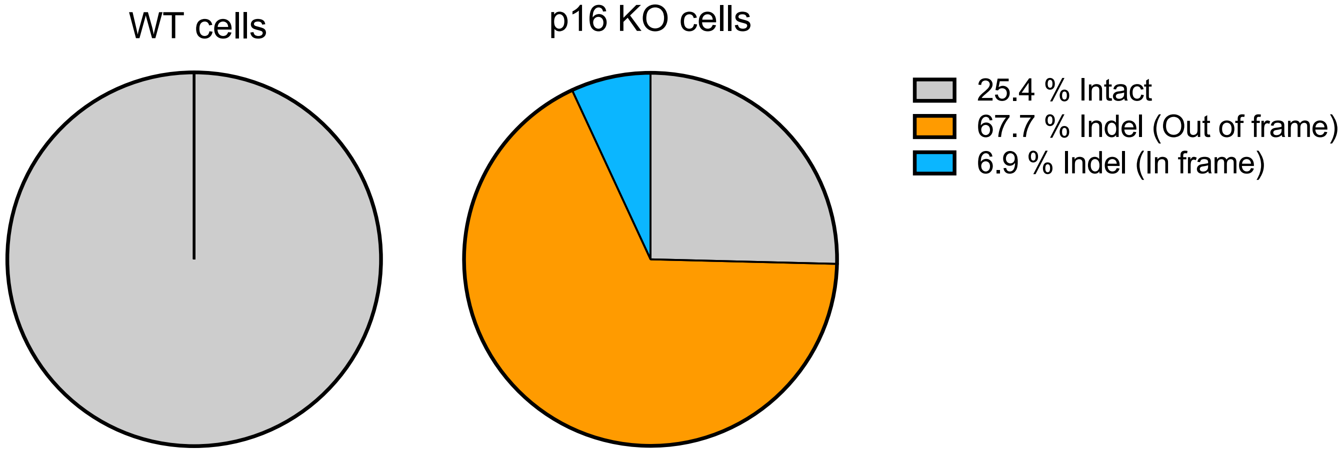

	PAM	
REFERENCE	CCTCTACCCACCTGGATCGGCCTCCGACCGTAACTATTCGGTGCGTTGGGCAGCGCCCCGCCTCCAGCAGCGCCCGCAC	
Intact		**25.4 %**
no indel	CCTCTACCCACCTGGATCGGCCTCCGACCGTAACTATTCGGTGCGTTGGGCAGCGCCCCGCCTCCAGCAGCGCCCGCAC	1145 reads
Indel (Out of frame)		**67.7 %**
1nt deletion	CCTCTACCCACCTGGATCGGCCTCCGACCGTAACTA_TCGGTGCGTTGGGCAGCGCCCCGCCTCCAGCAGCGCCCGCAC	702 reads
	CCTCTACCCACCTGGATCGGCCTCCGACCGTA_CTATTCGGTGCGTTGGGCAGCGCCCCGCCTCCAGCAGCGCCCGCAC	
1nt insertion	CCTCTACCCACCTGGATCGGCCTCCGACCGTAAACTATTCGGTGCGTTGGGCAGCGCCCCGCCTCCAGCAGCGCCCGCAC	
2nt deletion	CCTCTACCCACCTGGATCGGCCTCCGACCGTAA__ATTCGGTGCGTTGGGCAGCGCCCCGCCTCCAGCAGCGCCCGCAC	
4nt deletion	CCTCTACCCACCTGGATCGGCCTCCGACCGTAA____CGGTGCGTTGGGCAGCGCCCCGCCTCCAGCAGCGCCCGCAC	
	CCTCTACCCACCTGGATCGGCCTCCGACCGTA____TTCGGTGCGTTGGGCAGCGCCCCGCCTCCAGCAGCGCCCGCAC	
5nt deletion	CCTCTACCCACCTGGATCGGCCTCCGACCGTAAC_____GGTGCGTTGGGCAGCGCCCCGCCTCCAGCAGCGCCCGCAC	
Indel (In frame)		**6.9 %**
3nt deletion	CCTCTACCCACCTGGATCGGCCTCCGACCGTAA___TTCGGTGCGTTGGGCAGCGCCCCGCCTCCAGCAGCGCCCGCAC	316 reads

Supporting Figure S3
