# Supplementary material for: Highly efficient CRISPR-Cas9-mediated gene knockout in primary human B cells for functional genetic studies of Epstein-Barr virus infection
Source: PLoS Pathog. 2021 Apr 15;17(4):e1009117. doi: 10.1371/journal.ppat.1009117 (PMC8078793; doi:10.1371/journal.ppat.1009117)
Supplement: S1 Table — (DOCX) [file ppat.1009117.s006.docx]

**Supplementary Table 1. Results of 182 differentially expressed genes after 4sU-RNA labeling, isolation of newly transcribed RNA
and next generation sequencing of cDNA libraries**

| Gene | baseMean | log2FoldChange | lfcSE | stat | pvalue | padj |
| --- | --- | --- | --- | --- | --- | --- |
| AAK1 | 502.146800511834 | 0.81105148259538 | 0.227613896256642 | 3.56327753240906 | 0.000366253099247699 | 0.0329404123308122 |
| ACTB | 11997.9169487578 | -0.839045761904873 | 0.179186750064274 | -4.68252123331614 | 2.83367824322886e-06 | 0.00139109987757177 |
| ACTG1 | 5962.75929343749 | -0.680546763906181 | 0.180344854078576 | -3.77358570824354 | 0.00016091790133059 | 0.0205249820474698 |
| ANKRD36BP2 | 46.2257954700285 | -1.0044969497323 | 0.297362135325039 | -3.37802574841718 | 0.000730082442278297 | 0.0483248951400163 |
| ANTXR2 | 433.253294020029 | 0.698987597180395 | 0.20003651266505 | 3.49430005486453 | 0.000475306488768137 | 0.0378382503423391 |
| ARPC5 | 390.620257195093 | -0.720232868906817 | 0.192697062905163 | -3.73764321079084 | 0.000185753288930921 | 0.0210455459049929 |
| ASTN2 | 66.3270803502252 | 1.24715893149914 | 0.273286506085136 | 4.56355840383359 | 5.0293810226147e-06 | 0.00195020467131566 |
| ATPIF1 | 266.283012258013 | -0.886204154097208 | 0.253446128349792 | -3.49661744634788 | 0.00047119702442266 | 0.0377662812363794 |
| BACH2 | 2210.9553646691 | 0.804745304023015 | 0.182347420089095 | 4.41325302891488 | 1.01828867125437e-05 | 0.00315723082229448 |
| BCL11A | 1437.82719654409 | -0.684606858083214 | 0.174670135375898 | -3.91942707670038 | 8.87597115145582e-05 | 0.0137600910666385 |
| BCL2A1 | 956.448663293594 | -0.798482563126611 | 0.195188423241385 | -4.09082951676466 | 4.29833021637921e-05 | 0.00937831974247774 |
| BIRC5 | 38.2114126804302 | -1.01570990011471 | 0.295889846687202 | -3.43272981985239 | 0.000597537223387323 | 0.0434579232466015 |
| C14orf2 | 397.351662364471 | -0.866345194536922 | 0.231790528686408 | -3.73762120241337 | 0.00018576954456962 | 0.0210455459049929 |
| C1QBP | 585.635200940655 | -0.729863097250681 | 0.19228870769862 | -3.7956628134119 | 0.000147249520617436 | 0.0197147028626663 |
| C4orf46 | 94.2617637459468 | -0.906675982931393 | 0.249128675609516 | -3.63938828283467 | 0.000273286466590435 | 0.0270576567173824 |
| CACNA1A | 250.093395159451 | -0.83682714401574 | 0.247211153531635 | -3.38507034193607 | 0.00071160016370625 | 0.0480332231648256 |
| CAP1 | 1856.73746264745 | -0.586436353021153 | 0.159151877203509 | -3.68475925842377 | 0.000228918908532577 | 0.024298401624602 |
| CBR1 | 102.87483925602 | -0.90463245335937 | 0.252548295635724 | -3.58201765362223 | 0.000340950746378911 | 0.0316305645183963 |
| CCDC88B | 998.385259934577 | 0.918483488150147 | 0.184916195950526 | 4.96702564872081 | 6.79875989941295e-07 | 0.000616176839499103 |
| CCL3 | 1994.12976904047 | -0.782559555734676 | 0.197994338534624 | -3.95243400152993 | 7.73602694647926e-05 | 0.0126913996635382 |
| CCL4 | 496.498301626656 | -0.830576615348482 | 0.220932791089485 | -3.759408511759 | 0.000170315544285833 | 0.02090268482058 |
| CCNB1 | 103.095876086518 | -1.18270505441626 | 0.269083058135004 | -4.39531593929959 | 1.10611755674424e-05 | 0.00334160950091299 |
| CCNB2 | 54.8695991654698 | -1.06835488923503 | 0.287795489269055 | -3.71220164690017 | 0.000205464189019697 | 0.0228375384436799 |
| CCPG1 | 336.388335136036 | 0.879547875976597 | 0.22158799693481 | 3.96929386132479 | 7.20859377509809e-05 | 0.0124899488026773 |
| CD72 | 150.509572458527 | -0.887128588729041 | 0.251470948613926 | -3.52775775340561 | 0.000419095509346713 | 0.0352698806508784 |
| CD74 | 3787.17272361002 | -0.836327523273207 | 0.19887853627567 | -4.20521761138645 | 2.60831072391707e-05 | 0.00590983018253672 |
| CD79A | 459.917416397806 | -0.768656287667501 | 0.204651565607151 | -3.75592674010135 | 0.000172701211456229 | 0.0209769657049205 |
| CDKN2B | 24.2812924470959 | 1.00302118555144 | 0.295431631908489 | 3.39510423806658 | 0.000686024762491249 | 0.047842682071218 |
| CFL1 | 1033.54856443731 | -0.804705878742105 | 0.202715887440922 | -3.96962413208299 | 7.19860971995658e-05 | 0.0124899488026773 |
| CHI3L2 | 908.677766466595 | -0.781301825344857 | 0.21682512156561 | -3.60337316867797 | 0.000314114054654611 | 0.0295101636278342 |
| CKAP2L | 52.7570027242724 | -1.24460572697939 | 0.276872101738296 | -4.49523703964877 | 6.9492489523607e-06 | 0.0025586265986473 |
| CKS2 | 127.234200876141 | -0.975939019549939 | 0.257675280973644 | -3.78747629909352 | 0.000152185196562023 | 0.0199227331765973 |
| COX17 | 235.081312551411 | -0.81142686147219 | 0.231813731256459 | -3.50033993704409 | 0.000464665196547637 | 0.0374978448337278 |
| COX6A1 | 109.801998027521 | -0.966157577422555 | 0.256458185511774 | -3.76731035312655 | 0.000165015808970309 | 0.0205500444710553 |
| COX6C | 904.934133537843 | -1.01587067690667 | 0.228349843494721 | -4.44874698120894 | 8.63726905831179e-06 | 0.00282678622347304 |
| COX7C | 439.333986471741 | -1.12289933369674 | 0.231282085954084 | -4.85510725599243 | 1.20321600836547e-06 | 0.000945086067370795 |
| CYP1B1 | 73.0804561701443 | 1.1057402450702 | 0.279701383032146 | 3.95328844313621 | 7.70844196376086e-05 | 0.0126913996635382 |
| DCK | 609.782340798399 | -0.792908767847796 | 0.219570066146241 | -3.61118790809896 | 0.000304797716296921 | 0.0291961519789457 |
| DDB2 | 589.749088217455 | 0.750150272974449 | 0.197785029790616 | 3.79275556784348 | 0.000148984790660584 | 0.0197229079052022 |
| DNAH7 | 165.101051323602 | 1.66275454960592 | 0.269350615638158 | 6.17319750937435 | 6.69225235215983e-10 | 2.62827057377157e-06 |
| DUSP2 | 1165.03289335628 | -1.0355402427131 | 0.227124589112364 | -4.55934888758695 | 5.13124637674294e-06 | 0.00195020467131566 |
| EGR3 | 517.11458084048 | -0.919706605989208 | 0.201591424137774 | -4.56223080879002 | 5.06129634226025e-06 | 0.00195020467131566 |
| EID1 | 318.276281021312 | -0.753009520036842 | 0.214516802045171 | -3.510258930106 | 0.000447670530256045 | 0.0367800094849309 |
| EID3 | 280.389221389474 | 1.00732257636806 | 0.218460472526648 | 4.61100612260732 | 4.00724733853558e-06 | 0.00188555341578105 |
| ELL3 | 254.873625779832 | -0.871418902381822 | 0.21895328052779 | -3.97993078834559 | 6.89353351147187e-05 | 0.0124703472322331 |
| EXOC6B | 137.93198416506 | 0.791379505396459 | 0.235081531541955 | 3.36640441384577 | 0.000761549701330746 | 0.0495722573540268 |
| FAM100B | 331.480011515531 | -0.800159491724731 | 0.236828194138099 | -3.37864963517876 | 0.000728427783326129 | 0.0483248951400163 |
| FAM18B2 | 252.860915268992 | 0.925497648976995 | 0.237009684916913 | 3.9048937991772 | 9.42667730863509e-05 | 0.014424040526018 |
| FAM26F | 24.7948251958183 | -1.00215453873081 | 0.297514649158487 | -3.36842082084152 | 0.000756001082580834 | 0.0494844708609299 |
| FBXL20 | 366.041200928063 | 0.670468355755486 | 0.197486192768266 | 3.39501383037054 | 0.000686251338485473 | 0.047842682071218 |
| FBXO5 | 244.874103923622 | -0.799792123971707 | 0.226271724322999 | -3.53465341886915 | 0.000408310326593983 | 0.0352233609586013 |
| FLJ43663 | 1934.00893635379 | 0.688622216951205 | 0.168875023845861 | 4.07770315153139 | 4.548278433436e-05 | 0.00974323936413508 |
| GAPDH | 1790.09876682921 | -0.677616779869518 | 0.177917083132251 | -3.80860998809105 | 0.000139750181511081 | 0.0191457748670181 |
| GOLGA8A | 579.467702763301 | 0.634904499857189 | 0.182716217171577 | 3.47481197720391 | 0.000511211533093619 | 0.0392704675208476 |
| GPS2 | 859.44042557005 | -0.727639174509858 | 0.21479105678092 | -3.38766047997998 | 0.000704914652507322 | 0.0480332231648256 |
| H3F3B | 2296.839494682 | -0.525237814465021 | 0.156097245286879 | -3.36481155384726 | 0.00076595953781016 | 0.0495853586509852 |
| HDAC5 | 228.723036638826 | 0.800516508853198 | 0.216881909752442 | 3.69102480592752 | 0.000223352351744035 | 0.0242919975648105 |
| HIST1H1E | 783.987052779197 | -0.716322553838668 | 0.176477004920296 | -4.05901354775479 | 4.92804548003436e-05 | 0.0100107296285801 |
| HIST1H2AE | 251.872590780655 | -1.10475617433846 | 0.247657722873286 | -4.46081859076006 | 8.16471829896969e-06 | 0.00280890906530428 |
| HIST1H2AG | 259.839866706756 | -1.35602044270495 | 0.244740978257076 | -5.54063505164464 | 3.01376709854046e-08 | 4.43852549437546e-05 |
| HIST1H2AJ | 131.214776739578 | -1.23785337611661 | 0.29310036380166 | -4.22330890368412 | 2.40741499914508e-05 | 0.00567283270398546 |
| HIST1H2AL | 141.997619282872 | -1.18543554559933 | 0.291913888741794 | -4.06090834084252 | 4.88821610772026e-05 | 0.0100107296285801 |
| HIST1H2AM | 149.519836581144 | -0.992174906382489 | 0.284464533041282 | -3.48786857811376 | 0.00048688733484761 | 0.0381753838397577 |
| HIST1H2BB | 226.253736192257 | -1.3210370596314 | 0.277124155769054 | -4.76695023559153 | 1.8703548350233e-06 | 0.00107127022610895 |
| HIST1H2BC | 463.806262439231 | -1.3158076767577 | 0.236204172439584 | -5.5706368908206 | 2.53809805261672e-08 | 4.27198160799002e-05 |
| HIST1H2BD | 197.299087617718 | -0.902690170220389 | 0.22654676783899 | -3.98456433005456 | 6.76040417420825e-05 | 0.0124454815594565 |
| HIST1H2BF | 156.346028569642 | -1.10950798672697 | 0.262394733108712 | -4.22839274852094 | 2.35366667450385e-05 | 0.00567283270398546 |
| HIST1H2BH | 154.853244312484 | -1.04651412407681 | 0.29344713525956 | -3.56627820936519 | 0.000362086977362462 | 0.0328162212868041 |
| HIST1H2BI | 115.553839242361 | -0.99640041657222 | 0.260005054410889 | -3.83223479570362 | 0.000126984457420667 | 0.018470751571979 |
| HIST1H2BJ | 128.568289462335 | -0.986067945330725 | 0.245226502625674 | -4.02104966132436 | 5.79393875001882e-05 | 0.0111908502217577 |
| HIST1H2BK | 491.677332674824 | -0.950267070114055 | 0.213248168128037 | -4.45615584159907 | 8.34423843877522e-06 | 0.00280890906530428 |
| HIST1H2BL | 29.8059115792751 | -1.09728631579349 | 0.297411493974917 | -3.6894549740772 | 0.00022473499699239 | 0.0242919975648105 |
| HIST1H2BN | 93.0250184019872 | -0.949224512499467 | 0.258844729695562 | -3.66715796615171 | 0.000245261291306352 | 0.0256002811571811 |
| HIST1H3G | 106.756510819544 | -1.09116656538435 | 0.291359473833234 | -3.74508695745689 | 0.000180331304420274 | 0.0210362715710858 |
| HIST1H3H | 133.076944698674 | -1.06459387386129 | 0.270537744860122 | -3.93510293512544 | 8.31610461870459e-05 | 0.0130640459490103 |
| HIST1H4B | 252.971233961649 | -0.932477346145359 | 0.26741064359258 | -3.48706144833209 | 0.000488359136576773 | 0.0381753838397577 |
| HIST1H4C | 382.780388994244 | -0.986845841618361 | 0.255519952200395 | -3.86210874383857 | 0.000112412506294765 | 0.0169800531944221 |
| HIST1H4D | 116.648338488656 | -1.01840749917364 | 0.275282243261068 | -3.69950305224671 | 0.000216022042516774 | 0.0237866514479686 |
| HIST1H4F | 167.390306127519 | -1.42352104347865 | 0.292467835591283 | -4.86727383406355 | 1.13148201677934e-06 | 0.000945086067370795 |
| HIST2H2AB | 131.327808180982 | -1.36457954079195 | 0.266259440146999 | -5.1249996621287 | 2.97538279936115e-07 | 0.000389510668245257 |
| HIST2H2AC | 176.789112539706 | -1.00019542366697 | 0.251509128453514 | -3.97677583242006 | 6.98559597120511e-05 | 0.0124703472322331 |
| HIST2H2BF | 264.407629436371 | -1.33715654234754 | 0.219641166223301 | -6.0879140524509 | 1.14391247594131e-09 | 3.36939419788512e-06 |
| HIST2H3D | 62.4457446080419 | -1.03357552703225 | 0.277809687321541 | -3.7204445136428 | 0.000198872433100501 | 0.0223153810170486 |
| HMGB2 | 319.53753229559 | -0.834541249976746 | 0.230165320348696 | -3.62583402535374 | 0.000288030317181184 | 0.0280460594795761 |
| IDH3A | 918.271627875683 | -0.64527585994717 | 0.172574224155178 | -3.7391207354752 | 0.000184665025334631 | 0.0210455459049929 |
| IFI30 | 3089.30062936583 | -0.873902382486796 | 0.175520593707994 | -4.97891651358397 | 6.39412148768512e-07 | 0.000616176839499103 |
| IFIT3 | 6561.38552901699 | -0.989134220251223 | 0.222689004012747 | -4.44177396471091 | 8.92202566123908e-06 | 0.00284106233353294 |
| IFIT5 | 1368.59531531291 | -0.681476948459224 | 0.180672255997947 | -3.77189593773028 | 0.000162011825701468 | 0.0205249820474698 |
| IFITM2 | 90.2016104779524 | -1.00126646896262 | 0.282948437740881 | -3.53868880477637 | 0.000402119600411802 | 0.0350946157929767 |
| IGLL5 | 145.405055571413 | -1.27404945641496 | 0.270544205351546 | -4.7092099228644 | 2.48678850487989e-06 | 0.00127388444193456 |
| INADL | 370.249657184271 | 0.792577978430606 | 0.225859824468837 | 3.50915874611406 | 0.000449526512122733 | 0.0367800094849309 |
| ING3 | 783.994560183721 | -0.628884553306486 | 0.17592385100792 | -3.57475435936299 | 0.00035055686401991 | 0.0320175269138185 |
| IRF8 | 4919.39625197345 | -0.607500692113989 | 0.153793814524974 | -3.95009834426949 | 7.81190887671533e-05 | 0.0126913996635382 |
| IRS2 | 58.8975841877768 | 1.11512835893421 | 0.282649788924191 | 3.94526513951617 | 7.97117276440188e-05 | 0.0126913996635382 |
| KIAA0040 | 925.277902565187 | -0.676690305362049 | 0.184541260329178 | -3.66687809628586 | 0.000245529771750251 | 0.0256002811571811 |
| KIF2C | 130.736502420189 | -0.983089122771656 | 0.284861392335355 | -3.45111394251106 | 0.00055827782194978 | 0.0416305651785589 |
| LCP1 | 5032.11695095451 | -0.622397442358879 | 0.177277245224628 | -3.5108704536233 | 0.000446642000862554 | 0.0367800094849309 |
| LINC00152 | 201.776903684087 | 1.49694352278099 | 0.229216838186985 | 6.53068742515264 | 6.54685036294513e-11 | 3.85674954881097e-07 |
| LMO2 | 243.347717922205 | -0.958998976551315 | 0.278951624731224 | -3.43786840272155 | 0.00058631256328193 | 0.0429064262148305 |
| LOC100505812 | 128.947950490339 | -0.829701338672555 | 0.231926260363138 | -3.57743593749777 | 0.000346981222234091 | 0.0319385371903286 |
| LOC285758 | 76.0103868126759 | 0.952311354070453 | 0.271613876943576 | 3.50612186971685 | 0.000454686981249546 | 0.0369456690557389 |
| LOC646329 | 1319.71068471147 | 0.676698584930876 | 0.187859367848485 | 3.60215512636376 | 0.000315589935249287 | 0.0295101636278342 |
| LRRC32 | 86.0564290964123 | -1.04864185581951 | 0.274377065137039 | -3.82190054877859 | 0.000132427098796766 | 0.0187982660002832 |
| LSM5 | 238.729336521996 | -0.932575062408776 | 0.248851322837016 | -3.74751900764282 | 0.000178592307055826 | 0.0210362715710858 |
| LSM6 | 276.774501482351 | -0.956823224583512 | 0.233658999826412 | -4.09495557754825 | 4.2224934112276e-05 | 0.00937831974247774 |
| LTA | 105.09165559495 | -1.50062861074605 | 0.251421603695061 | -5.96857465186685 | 2.39335137271227e-09 | 5.6396931746592e-06 |
| LY6E | 639.811187102905 | -0.938467921580419 | 0.231736160986379 | -4.04972585023354 | 5.12776689052255e-05 | 0.0101240989421758 |
| LYSMD2 | 444.778556218072 | -0.718958384376552 | 0.197504174739399 | -3.64021867044176 | 0.000272406598329968 | 0.0270576567173824 |
| MAD2L1 | 44.4208564832488 | -1.04428765585321 | 0.288950659775489 | -3.6140691170756 | 0.00030142864980939 | 0.0291101012463462 |
| MAP2K6 | 72.7678006687341 | -1.01863382202314 | 0.276293057067865 | -3.68678761903501 | 0.000227102736424203 | 0.024298401624602 |
| MARCKSL1 | 1041.40060549034 | -0.652352574263795 | 0.191441761798256 | -3.40757715629077 | 0.000655423755423564 | 0.0462407346491044 |
| MIF | 220.760403549176 | -0.955094734457697 | 0.26940755512616 | -3.54516685328457 | 0.000392364628941417 | 0.0344988064043864 |
| MRPS18B | 36.0178059601725 | -1.26101263883435 | 0.298374945934295 | -4.22626851220961 | 2.37598454019713e-05 | 0.00567283270398546 |
| MRPS22 | 424.730057110574 | -0.667416696908318 | 0.197332680808994 | -3.38219039123244 | 0.000719102867682549 | 0.0481390340172488 |
| MT2A | 31.1901138830021 | -1.13789671782981 | 0.29822008304418 | -3.81562739240884 | 0.000135837352549006 | 0.018960957500115 |
| MYD88 | 518.399946998398 | -0.686161942464106 | 0.200775110771641 | -3.41756475604458 | 0.000631840611741318 | 0.0453305392198682 |
| MYL12A | 1344.64905296711 | -1.15263317485607 | 0.194485949020083 | -5.92656271912499 | 3.09341269285814e-09 | 6.0744313912091e-06 |
| N4BP2 | 573.907931277517 | 0.678129129115032 | 0.192074545535983 | 3.53055178250047 | 0.000414693832198394 | 0.0352698806508784 |
| NAB2 | 349.53733734384 | -0.795098966229857 | 0.228388416539025 | -3.48134541269082 | 0.000498901669340115 | 0.0386714438695081 |
| NACA | 4515.66311559427 | -0.811542672085577 | 0.213352566103936 | -3.80376335239497 | 0.00014251433223539 | 0.019300044395372 |
| NCF4 | 239.768485741895 | -0.76883644484025 | 0.221358370398186 | -3.47326574304484 | 0.00051416598876556 | 0.0392704675208476 |
| NFKBIE | 846.209251285846 | -0.824046286289688 | 0.188663379380606 | -4.36781260356454 | 1.25497099078845e-05 | 0.00350045257636285 |
| NLRP4 | 16.5105665004251 | -1.00815985003181 | 0.293052110430064 | -3.44020675555723 | 0.000581269955210167 | 0.0429064262148305 |
| NUSAP1 | 163.485066615911 | -0.920312926389469 | 0.26085437190661 | -3.52807169633698 | 0.000418598760756948 | 0.0352698806508784 |
| PDE4B | 2224.27728943231 | 0.778687706112849 | 0.177848123874246 | 4.37838583365349 | 1.19561544917523e-05 | 0.00343579054199575 |
| PFN1 | 823.085189212955 | -0.870464751058442 | 0.204655091606931 | -4.25332565255836 | 2.106189339443e-05 | 0.00539459191246031 |
| PGAM1 | 337.226162185102 | -0.664394933129412 | 0.194863350974664 | -3.40954278886334 | 0.000650718692221878 | 0.046185347179266 |
| PNPLA7 | 169.097154429514 | 1.08036707766161 | 0.236950304895805 | 4.55946692339849 | 5.12836331804196e-06 | 0.00195020467131566 |
| POLE3 | 1200.81973471043 | -0.646075828914661 | 0.191685298607071 | -3.37050276473748 | 0.000750311531025356 | 0.0493864271426857 |
| PRDM8 | 102.661310978545 | 1.24198523765087 | 0.283000699004702 | 4.38862957589454 | 1.14067164837861e-05 | 0.00335984834029918 |
| PSMA4 | 526.255802553606 | -0.69947663312964 | 0.191895207208556 | -3.6450969427779 | 0.000267291026072576 | 0.0270576567173824 |
| PSME1 | 2037.73774019224 | -1.01270942930033 | 0.220002310648379 | -4.60317633172001 | 4.16095644290506e-06 | 0.00188555341578105 |
| PSME2 | 971.180692180407 | -0.672101456803177 | 0.179316729972816 | -3.74812465577008 | 0.000178161707284455 | 0.0210362715710858 |
| PTMA | 6636.84165589177 | -1.03722007007572 | 0.217929836333445 | -4.75942205769713 | 1.94148078989883e-06 | 0.00107127022610895 |
| PTPN6 | 2337.6398778381 | -1.19322806289378 | 0.171083378957887 | -6.97454112820335 | 3.06870764522717e-12 | 3.61555134760665e-08 |
| PVT1 | 1859.42929391284 | 0.746288295367704 | 0.17738889122859 | 4.20707458172231 | 2.58697782724832e-05 | 0.00590983018253672 |
| RAC2 | 667.740002987227 | -0.793479469358242 | 0.194918826720946 | -4.07082005728579 | 4.68479273624068e-05 | 0.00985646928899781 |
| RANBP1 | 898.622742041506 | -0.708941153174138 | 0.204984330957982 | -3.45851387694339 | 0.00054316429085337 | 0.0410228312489385 |
| RAPGEF5 | 296.411819898576 | -0.861008392792227 | 0.252030923809607 | -3.41628074752709 | 0.000634827615963186 | 0.0453305392198682 |
| RASSF6 | 248.41618383936 | -0.926996009809418 | 0.21657954947371 | -4.28016408780065 | 1.86755569038632e-05 | 0.00500080480548445 |
| RBM7 | 536.22892806372 | -0.649266953236267 | 0.18880651363508 | -3.43879530814891 | 0.000584308859014945 | 0.0429064262148305 |
| RPL14 | 1188.99009679804 | -0.831799297929183 | 0.233741454412551 | -3.55862976903133 | 0.000372794590096648 | 0.0332747413675659 |
| RPL19 | 2452.36495792944 | -0.878135194057389 | 0.21896978988057 | -4.01030294880559 | 6.06408911193337e-05 | 0.0113408091931427 |
| RPL22 | 1807.83808807415 | -0.850839619013236 | 0.221541908292072 | -3.8405357504257 | 0.000122766091357926 | 0.0180803761047385 |
| RPL23A | 2771.3903382821 | -0.970677536271651 | 0.22730877519413 | -4.27030384305514 | 1.95206843643126e-05 | 0.00511094895956291 |
| RPL35 | 1007.35201145352 | -0.886077620838956 | 0.223707820540014 | -3.96087011486693 | 7.46771467033854e-05 | 0.0126913996635382 |
| RPL36AL | 1446.77351367091 | -1.07603176457324 | 0.214675234782638 | -5.01237027020251 | 5.37636216799169e-07 | 0.000575857264211619 |
| RPL39 | 368.347912460676 | -0.841707508251418 | 0.242428461601283 | -3.47198304477861 | 0.000516628965008605 | 0.0392704675208476 |
| RPL8 | 1081.5371919421 | -0.765148555364089 | 0.21203088989748 | -3.60866549083507 | 0.000307776155487306 | 0.0292436989028342 |
| RPS20 | 876.939506713842 | -0.791910802905722 | 0.207638159570499 | -3.81389819936661 | 0.000136791833942435 | 0.018960957500115 |
| SAT1 | 2083.10941075602 | -1.06844339971585 | 0.234029706402477 | -4.56541785288732 | 4.98500379874219e-06 | 0.00195020467131566 |
| SBF2 | 341.499545555033 | 0.959081876518784 | 0.198625620630363 | 4.82859096160415 | 1.37502522228294e-06 | 0.000952973362878684 |
| SEMA4A | 161.796451176425 | -0.827883803744029 | 0.242150310197744 | -3.41888392820131 | 0.000628785426395743 | 0.0453305392198682 |
| SERPINA9 | 129.64906401049 | -1.38634910369716 | 0.275126668000718 | -5.03894847333934 | 4.6809647325116e-07 | 0.000551511264784517 |
| SLC25A5 | 455.472465792395 | -0.714874354611858 | 0.21122885184652 | -3.38435942042279 | 0.000713445429794983 | 0.0480332231648256 |
| SLC7A5P2 | 1208.10955376166 | 0.669971020347771 | 0.197698708578276 | 3.38884874446464 | 0.000701867146021403 | 0.0480332231648256 |
| SMN1 | 66.816853106975 | 1.20447102317806 | 0.297513839206782 | 4.04845376735875 | 5.15571156450981e-05 | 0.0101240989421758 |
| SMS | 271.610550202915 | -0.782704205224778 | 0.214685798395588 | -3.64581267635849 | 0.000266548096087216 | 0.0270576567173824 |
| SNRPD1 | 1817.9640448995 | -0.852610880133532 | 0.226380116630894 | -3.76627988722035 | 0.000165698032995268 | 0.0205500444710553 |
| SNRPF | 443.579939164936 | -0.855241178921422 | 0.245296031358336 | -3.48656753305585 | 0.000489261836683366 | 0.0381753838397577 |
| SNX29 | 1536.83336607446 | 0.604853659327698 | 0.171682133657752 | 3.52310194684248 | 0.000426527261098301 | 0.0356407389380155 |
| SSTR2 | 58.1229921855203 | -1.32278530097286 | 0.276745732953237 | -4.77978571469566 | 1.7548212839925e-06 | 0.00107127022610895 |
| STARD9 | 485.753289396133 | 0.920327780057352 | 0.193615017354028 | 4.75339047887239 | 2.00033483062272e-06 | 0.00107127022610895 |
| SULF2 | 79.7400183226873 | 0.948282130100853 | 0.260204110269327 | 3.64437798126757 | 0.000268039260572515 | 0.0270576567173824 |
| SUMO3 | 229.916253262554 | -0.801854965592115 | 0.220674418556968 | -3.63365618378241 | 0.000279433167346964 | 0.0274356798140161 |
| SYTL1 | 151.474705236325 | 0.807426477274001 | 0.233601375039789 | 3.45642861535586 | 0.000547384170091364 | 0.041078218420487 |
| TBCC | 264.722721605275 | -0.834672346169106 | 0.211400516477569 | -3.94829851921232 | 7.87086193031726e-05 | 0.0126913996635382 |
| TCEAL8 | 79.6295001769609 | -1.04206668656962 | 0.278230086779901 | -3.7453414856387 | 0.000180148565084504 | 0.0210362715710858 |
| TCL1A | 218.209618319557 | -0.716596805265575 | 0.211703982100448 | -3.38489998230439 | 0.000712041944292738 | 0.0480332231648256 |
| TMCO7 | 658.065094034215 | 0.640798258331005 | 0.180480634233505 | 3.55050978767032 | 0.000384485838876204 | 0.0340602417566875 |
| TMEM163 | 98.9223447898854 | 1.27578324239639 | 0.264105902804153 | 4.83057451140138 | 1.36139654458101e-06 | 0.000952973362878684 |
| TMOD2 | 594.759564074376 | -0.705070006680488 | 0.183153493693948 | -3.84961265253651 | 0.000118304763726826 | 0.0176438826104996 |
| TMSB4X | 2316.1271528341 | -0.880717548996553 | 0.197390199671251 | -4.46180990983022 | 8.12703041243315e-06 | 0.00280890906530428 |
| TNRC6C | 308.94320768588 | 0.848897314180509 | 0.22181128731705 | 3.82711504201823 | 0.0001296539280583 | 0.0186290558583279 |
| TPI1 | 1327.6777822795 | -0.743553598481237 | 0.204304334268063 | -3.63944113640603 | 0.000273230384332824 | 0.0270576567173824 |
| TPM4 | 2667.98949854888 | -0.574169423192046 | 0.169308368305863 | -3.39126428857186 | 0.000695709777799284 | 0.0480332231648256 |
| TREX1 | 279.834085798904 | -0.937262981287969 | 0.214775593400891 | -4.36391754969343 | 1.27753743662878e-05 | 0.00350045257636285 |
| TUBA1B | 1581.504777123 | -0.710409516613346 | 0.201030654859484 | -3.53383675295644 | 0.000409573964634899 | 0.0352233609586013 |
| UQCR10 | 82.0448989996799 | -1.06979753136939 | 0.266525175510863 | -4.01387046953013 | 5.97311335016572e-05 | 0.0113408091931427 |
| VAV3 | 690.029972043826 | 0.758282267713259 | 0.178872418949934 | 4.23923527263026 | 2.24282505865426e-05 | 0.00562233294490733 |
| ZDHHC14 | 349.764024644189 | 0.939459674678294 | 0.195734617799963 | 4.79966030147208 | 1.58934979418156e-06 | 0.00104031773750262 |
| ZFYVE9 | 122.428441432806 | 0.988088407950424 | 0.261705622965163 | 3.77557194513148 | 0.000159640937462919 | 0.0205249820474698 |
