# Supplementary material for: Highly efficient CRISPR-Cas9-mediated gene knockout in primary human B cells for functional genetic studies of Epstein-Barr virus infection
Source: PLoS Pathog. 2021 Apr 15;17(4):e1009117. doi: 10.1371/journal.ppat.1009117 (PMC8078793; doi:10.1371/journal.ppat.1009117)
Supplement: S2 Table — (PDF) [file ppat.1009117.s007.pdf]

**Supplementary Table 2. crRNA sequences**

| Target gene | IDT design ID | Naming in the text | Nucleotide sequence (5' - 3') | PAM sequence | Purpose |
|---|---|---|---|---|---|
| CD46 | Hs.Cas9.CD46.1.AA | CD46-gRNA 1 | TCGTTCACCAATCTCATAGT | AGG | CRISRP-Cas9 technology |
| CD46 | Hs.Cas9.CD46.1.AD | CD46-gRNA 2 | TTTGTGATCGGAATCATACA | TGG | CRISRP-Cas9 technology |
| CDKN2A | Hs.Cas9.CDKN2A.1.AA | CDKN2A-gRNA | CCCAACGCACCGAATAGTTA | CGG | CRISRP-Cas9 technology |
