# Supplementary material for: Highly efficient CRISPR-Cas9-mediated gene knockout in primary human B cells for functional genetic studies of Epstein-Barr virus infection
Source: PLoS Pathog. 2021 Apr 15;17(4):e1009117. doi: 10.1371/journal.ppat.1009117 (PMC8078793; doi:10.1371/journal.ppat.1009117)
Supplement: S3 Table — (PDF) [file ppat.1009117.s008.pdf]

Supplementary Table 3. List of Primer pairs				
No	Gene	Strand	Nucleotide sequence (5' - 3')	Purpose
1	CD46	forward	CTGTACTACCTGCTGCCAGACC	Gel electrophoresis
2	CD46	reverse	ATAACAGGCGTCATCTGAGACAGG	Gel electrophoresis
3	CD46	forward	ACACTCTTTCCCTACACGACGctcttccgatctCTGTACTACCTGCTGCCAGACC	Miseq-single end seq
4	CD46	reverse	TGACTGGAGTTCAGACGTGTGctcttccgatctATAACAGGCGTCATCTGAGACAGG	Miseq-single end seq
5	CD46	forward	ACACTCTTTCCCTACACGACGctcttccgatctGACCACAGTCCATGGCTGATG	Miseq-paired end seq
6	CD46	reverse	TGACTGGAGTTCAGACGTGTGctcttccgatctCATCACCGTAGTGGAATATGTACCC	Miseq-paired end seq
7	CDKN2A	forward	ACACTCTTTCCCTACACGACGctcttccgatctCGTCCTCCAGAGTCGCCC	Miseq-single end seq
8	CDKN2A	reverse	TGACTGGAGTTCAGACGTGTGctcttccgatctCTGCGGAGAGGGGGAGAG	Miseq-single end seq
