# Supplementary material for: Highly efficient CRISPR-Cas9-mediated gene knockout in primary human B cells for functional genetic studies of Epstein-Barr virus infection
Source: PLoS Pathog. 2021 Apr 15;17(4):e1009117. doi: 10.1371/journal.ppat.1009117 (PMC8078793; doi:10.1371/journal.ppat.1009117)
Supplement: Attachment — Submitted filename: PLOS Pathogens_point-by-point.docx [file ppat.1009117.s009.docx]

**Research Unit Gene Vectors**

Prof. Dr.

**Wolfgang Hammerschmidt**

Head of Unit

Phone	+ 49 (0) 89 3187 1506

Fax	+ 49 (0) 89 3187 4225

hammerschmidt@helmholtz-muenchen.de

http://www.helmholtz-muenchen.de/en/agv/

February 28^th^ , 2021

Helmholtz Zentrum München

German Research Center for

Environmental Health

Ingolstädter Landstrasse 1

85764 Neuherberg

Phone + 49 (0) 89 3187 (0)

Fax	+ 49 (0) 89 3187 3322

www.helmholtz-muenchen.de

Aufsichtsratsvorsitzende:

MinDir’in Prof. Dr. Veronika von Messling

Geschäftsführer:

Prof. Dr. med., Dr. h.c. Matthias Tschöp

Kerstin Günther

Registergericht:

Amtsgericht München HRB 6466

USt-IdNr. DE 129521671

Bankverbindung:

Münchner Bank eG

Konto-Nr. 2 158 620

BLZ	701 900 00

IBAN	DE04 7019 0000 0002 1586 20

BIC	GENODEF1M01

**Subject: Manuscript resubmission to PLOS Pathogens**

**Response to Reviewers**
**Response to Reviewers**

We thank the three expert Reviewers for their appreciation of our work and for their suggestions to improve our manuscript further. Please find below point-by-point answers to all comments and suggestions. The Reviewers’ questions and comments are in black, our answers in blue.

**Part I - Summary**

Reviewer #1: In the work by Akidil and colleagues, a method was established to perform highly efficient CRISPR-Cas9-mediated gene knockout in primary human B cells. Nucleofection of B cells with preformed Cas9 ribonucleoprotein complexes was followed by EBV-mediated immortalization of the transfected B cells. Knockout of the CD46 gene was used as a model. This method was then applied to study an aspect of EBV infection of B cells. It is shown that EBNA3C has an important role in downregulating CDKN2A in lymphoblastoid cell lines, confirming earlier indications in this direction.

As primary B cells are notoriously difficult to genetically modify, the present novel protocol is a valuable tool to generate CRISPR-Cas9-modified cells. The manuscript is overall well written and the data are clearly presented.

The EBV infection of the cells as an intrinsic/mandatory part of the method is a restriction of the method and should be clearly mentioned already in the Abstract and Author summary.

We thank Ralf for his appreciation of our work. As suggested, Abstract and Author summary were altered such that the requested information (‘EBV infection of the cells’) is now provided. We would like to restate that infection with EBV is only needed to ensure survival of the primary B cells. As a matter of fact, infection is not an intrinsic or obligatory part of our protocol. As shown in Figure 2A and in the new panel of Supplementary Figure 2D CRISPR-Cas9 mediated editing of the *CD46* locus is very efficient in non-infected cells (Fig. 2A) and in B cells propagated on CD40 ligand feeder cells together with IL-4 (Suppl. Fig. 2D) to maintain and expand the non-infected B cells *in vitro*.

Reviewer #2: The authors established a method and characterized its efficacy as well as kinetics to insert gene deletions via CRISPR/Cas9 in primary human B cells. They initially targeted CD46 with their knock-out technology and then CDKN2A, the gene for the CDK4 inhibitor p16INK4a. p16INK4a is in addition to BLIMP-1 one of the main targets for suppression by EBNA3C. Accordingly, the authors tested if p16INK4a deficient B cells got enriched during B cell transformation by deltaEBNA3C EBV infection towards lymphoblastoid cell lines (LCLs). This was indeed the case, but deltaEBNA3A/C deficient EBV did not induce significant plasma cell differentiation in the absence of p16INK4a. With these experiments the authors elegantly combined genetic host gene knock-outs and recombinant EBV mutants to conclusively address the dependence of EBV on EBNA3C mediated p16INK4a suppression during B cell transformation.

Even so p16INK4a had previously been identified as a target of EBNA3C, the reported experiments suggest p16INK4a inhibition as the main function of EBNA3C during B cell transformation by EBV, with CDKN2A knock-out cells getting enriched during deltaEBNA3C EBV infection to more than 80% in a competitive outgrowth assay. It would, however, be interesting to report what happens to the other main target BLIMP-1 in this EBNA3C deficient B cell transformation.

We thank this Reviewer for his or her appreciation of our contribution. From a general point of view, we made use of *CDKN2A* because work by Martin Allday’s group suggested that a knockout of *CDKN2A* by Cas9 ribonucleotide complexes can lead to a gain-of-function situation, which was handy to demonstrate the power of gene editing in primary B cells. It was not our foremost intention to study B cell differentiation using the CRISPR-Cas9 technology.

Reviewer #3: In this study, Akidil et al present an exciting technical development for efficient gene knockout in primary human B cells. They use CD46 as a test case initially and then move to more biologically relevant targets including the cyclin-dependent kinase inhibitor, p16INK4A. The innovation here is to use the Cas9/guide RNA ribonucleoprotein (RNP) complex to transfect primary resting human B cells followed by EBV infection to activate and induce proliferation of these cells. Initial studies support efficient knockout (KO) of the cell surface marker, CD46, which is easy to monitor by flow cytometry. They find that one guide is capable of ~60% KO by flow cytometry and sequencing of the target genomic DNA site while two guides approaches 80% KO. Temporal analysis of allele targeting indicates progressive error-prone repair through the first 24h after transfection with the highest indel frequencies reported at 4 days post transfection and persistent through 8 days. Following the fate of the RNAs in these (CD46 sg1+2) cells indicate about 50% of the CD46 transcripts contain indels or deletions. However, this transfection did not significantly impact B cell gene expression, as anticipated.

To follow up with a biologically relevant target, the authors transfect RNPs against p16INK4a, which the viral EBNA3 proteins are known to antagonize. Excitingly, this novel approach recapitulated the findings by the Allday group indicating that p16 loss enabled outgrowth of B cells infected with an EBNA3C deleted EBV. The follow up to this experiment was a test of the recent hypothesis that the loss of both EBNA3A and 3C leads to a plasma cell phenotype with increased expression of CD138 (proxy for antibody secretion) and CD38 (activation marker). The data presented support this hypothesis, though the phenotype was not as strong as shown by Styles, et al. There are technical challenges associated with observing a true differentiation to the plasma cell lineage and antibody secretion. For example, these cells will likely cease to proliferate. However, with p16 knocked-out, the authors conclude that this terminal differentiation of 3A/3C KO infected B cells does not occur. It may be of interest to determine Ig expression and secretion is increased in these p16KO, EBNA3A/3C KO cells at 20 days (or earlier) post infection.

We thank Micah Luftig for his thoughtful considerations and the appreciation of this technological breakthrough. Our working with CDKN2A/p16INK4a was opportunistic because we could predict the outcome and phenotype of its knockout given Martin Allday’s findings. We are happy that our experiments recapitulated the role of EBNA3C in inactivating *CDKN2A*, but we did not want to move into the area of terminal B cell differentiation. As Micah pointed out in his comment above, this is difficult territory. As one of the consequences, we did not measure antibody levels in the supernatants of the different B cell cultures, because conventional, wildtype EBV infected LCLs can release substantial amounts of immunoglobulins (Lee and Sugden. 2008. The LMP1 oncogene of EBV activates PERK and the unfolded protein response to drive its own synthesis. *Blood*. 111:2280-2289. doi:10.1182/blood-2007-07-100032). One should also not forget that the spliced form of XBP-1 plays an important role in differentiation of B cells to plasma cells and immunoglobulin synthesis, an aspect that is likely not a direct consequence of knocking out *CDKN2A* or of deregulating CDKN2C/p18INK4c. As pointed out in our comment regarding Reviewer #2 and his or her first point of critique, we maintain that the very moderate phenotypes shown in Figure 4D in our manuscript make it very unlikely that a detailed analysis of secreted immunoglobulins (per cell and per hour for the many cell lines including replicates) is a worthwhile addition. We are happy to provide the EBV producer cell lines if colleagues with interest in B cell differentiation of EBV-infected cells find this aspect more appealing (and rewarding).

**Part II – Major Issues: Key Experiments Required for Acceptance**

Reviewer #1: No additional experiments requested.

Reviewer #2: 1. The authors report minimal plasma cell surface marker up-regulation during CDKN2A deficient B cell transformation. It would be interesting to report the BLIMP-1 levels in the knock-out and mutant infected B cells. BLIMP-1 is considered as one of the main transcription factors for plasma cell differentiation.

We agree with this Reviewer’s statement, but we did not wish to move into this direction. This is because it was not our intention to study terminal B cell differentiation. As Micah Luftig (Reviewer #3) pointed out terminally differentiated plasma cells will likely cease to proliferate, which could prevent their accumulation in these probably heterogeneous cell populations. Systematic transcriptional profiling of mature (murine) B cells up to plasma cells revealed that Blimp1/PRDM1 and Syndecan 1/SDC1 transcript levels increased 60-fold starting with follicular B cells to splenic plasma cells (PCs) (Shi et al. 2015. Transcriptional profiling of mouse B cell terminal differentiation defines a signature for antibody-secreting plasma cells. *Nat Immunol*. 16:663-673. doi:10.1038/ni.3154). At first glance this is what one expects but the situation is more complex. Both genes are already highly transcribed in lymphoblasts such that the final “step” of PC differentiation is accompanied by a two- to three-fold increase of Blimp1 transcripts at best in an *in vivo* situation (in mice). Given the very modest phenotypes shown in Figure 4D in our manuscript, we think that the expected delta in Blimp1 levels in our model will be too small to be informative.

2. The authors seem to have used EBNA3A and EBNA3C double-deficient EBV for the transformation of CDKN2A deficient B cells to investigate plasma cell differentiation, while they used EBNA3C deficient EBV for the competitive wt versus CDKN2A knock-out infection experiments. Is there a particular reason for this? Would plasma cell differentiation be enhanced in the presence of EBNA3A?

The study by Martin Allday’s laboratory revealed that EBV-infected lymphoblastoid B cells differentiated spontaneously and acquired a plasma cell-like phenotype in the absence of both EBNA3A and EBNA3C (Styles et al., 2017). Their findings also revealed that a non-functional EBNA3C allele in the presence of a functional, wildtype EBNA3A gene did not relieve repression of Blimp1 (Fig. 4A in Styles et al., 2017). In contrast, the regulation of p16INK4a/CDKN2A is solely dependent on EBNA3C (our manuscript). This observation led us to study plasma cell differentiation in *CDKN2A* knockout cells infected with our EBNA3A and EBNA3C double-negative mutant EBV (Fig. 4D) to promote maximal plasma cell differentiation.

3. It would be good if a summary graph could be provided for the four experiments of figures 4B and S3A with statistical evaluation. Maybe enrichment of CDKN2A indel B cells in deltaEBNA3C versus wt EBV infection could be compared at select timepoints.

We thank this Reviewer for his or her idea and suggestion. Consequently, we performed linear regression analyses on data from these four experiments to calculate the slopes of cell samples infected with WT EBV or ΔEBNA3C EBV (Supplementary Fig. S4A). Slopes were transferred into a column data table and the p-value (unpaired t-test) provided a convincing level of significance (p<0.05; Supplementary Fig. S4B).

Reviewer #3: (No Response)

**Part III – Minor Issues: Editorial and Data Presentation Modifications**

Reviewer #1: a) A major aspect of the method is that one day after nucleofection the cells are infected with EBV to immortalize them. Thus, although the experiment is indeed started with primary B cells, one later obtains EBV-immportalized lymphoblastoid cell lines. As EBV has a massive influence on B cell physiology and gene expression, this is an important aspect/caveat of the method. Therefore, the EBV infection of the cells as an intrinsic/mandatory part of the method should be clearly mentioned already in the Abstract and Author summary.

As already mentioned in Part I of this review, we used EBV infection to ensure long-term B cell survival as a convenient measure. In the first version of our manuscript, we showed that an almost complete knockout of *CD46* is feasible in non-infected primary B cells (Fig. 2A). Given this Reviewer’s concern, we added a new experiment in which we cultivated and expanded primary B cells on irradiated CD40L feeder cells together with IL-4 after nucleofection with *CD46* specific RNP complexes. The very convincing outcome is now provided in Supplementary Figure S2D.

b) From the Methods section it becomes obvious that the studies were not performed with pure B cells, but with tonsillar mononuclear cells that were depleted from T cells by erythrocyte rosetting and cyclosporin A treatment. Hence, the resulting cells likely also encompass monocytes, NK cells, and other cells present in tonsils. Did the authors test the B cell fraction in the suspensions they used? Could it be that the non-B cells present in the suspensions used could have a supporting role in the analysis? This would be relevant to know if others plan to use the method for isolated, pure B cells.

Our studies with B cells isolated from adenoid tissue revealed a minute fraction of non-B cells as shown in panel A of the newly added Supplementary Figure S2. We do not think that these cells contribute anything to the success of genetic manipulation or subsequent expansion the B cells. This Reviewer’s comment pointed us to an error in our Materials and Methods section, because we used Ciprobay (an antibiotic to ensure sterility of the cells after isolation and subsequent nucleofection) but we wrote cyclosporin A. We thank this Reviewer for taking note. This error is now corrected in the revised manuscript.

c) The cells from adenoids that were used encompass naive B cells, memory B cells, germinal center B cells, and a few transitional and CD5-positive mature B cells. Do the authors have any data whether all of these B cell subsets are well nucleofected and genetically modified? As germinal center B cells activate an apoptosis program once they are in suspension, it would be interesting to know whether also these cells can be modified and rescued by the approach.

We know from previous studies that EBV infection and reprogramming of activated germinal center B cells is very inefficient compared with resting naïve or memory B cells isolated from adenoid biopsies (Kalla et al. 2010. AP-1 homolog BZLF1 of Epstein-Barr virus has two essential functions dependent on the epigenetic state of the viral genome. *Proc Natl Acad Sci U S A*. 107:850-855. doi:10.1073/pnas.0911948107). Given this biological or experimental difficulty we did not look specifically at germinal center B cells in this study. We are convinced, however, that they, too, should be amenable to this technology.

d) Although the method as such is novel, the authors should also refer to and briefly discuss two other recent methods for CRISPR-Cas9-mediated gene knockout in primary human B cells: R. Caeser et al., Nature Commun 2019 (focussed on germinal center B cells), M.J. Johnson et al., Scientific Reports, 2018.

We corrected this mishap. Our revised manuscript includes a brief discussion of these two recent studies in Discussion and Introduction.

e) The authors used cyclosporin A in the initial culture. This was likely done to suppress T cells. However, as cyclosporin A affects NFAT signaling, which has also been reported to occur in some circumstances in B cells, did the authors consider potential effects of cyclosporin A on subsets among their mixed B cell populations?

We are aware that cyclosporin A might cause such effects in development of cells but there was an error from our side in Materials and Methods stating that cyclosporin A has been used throughout the experiments. We did not use cyclosporin A but rather the antibiotic Ciprobay to prevent possible bacterial contamination resulting from surgery and nucleofection.

f) The numbering of the supplementary tables is not in chronological order.

This has been corrected in Supplementary Tables and Figures throughout the revised manuscript.

g) The oligonucleotide sequences in supplementary tables 1 and 2 should be provided with information on their orientation (mark 5‘ and 3‘ ends).

We corrected this issue in the two Supplementary Tables.

h) In the legend to Supplementary figure S3, the reference to main figure 3 is likely wrong.

We corrected this error in the legend of Supplementary Fig. S3, which is now Supplementary Figure S4 in the revised manuscript.

Reviewer #2: 1. The timepoint of analysis after EBV infection could be added for clarity to figure 1A.

In Figure 1A, we did not include a clear statement when the (EBV-infected) cells were analyzed as we wanted to introduce a general scheme of the protocol. In addition, the time points of analyses varied between different types of experiments. Most individual experiments used different time points, which are specifically mentioned in figure legends, such as in Figure 1B and Figure 2, for example.

Reviewer #3: (No Response)
